# Xylose and cellobiose fermentation by yeasts isolated from the Brazilian biodiversity

**DOI:** 10.1186/1753-6561-8-S4-P202

**Published:** 2014-10-01

**Authors:** Adriane Mouro, Raquel M Cadete, Renata O Santos, Carlos A Rosa, Boris U Stambuk

**Affiliations:** 1 Universidade Federal de Santa Catarina, Departamento de Bioquímica, Florianópolis, SC, Brazil; 2 Universidade Federal de Minas Gerais, Departamento de Microbiologia, Belo Horizonte, MG, Brazil

## Background

The production of fuel ethanol has become important in recent years due not only to the future depletion of fossil fuels, but also environmental concerns. An attractive source of raw material for ethanol production is the lignocellulosic biomass, composed of lignin, cellulose and hemicellulose. In the case of Brazil, the sugarcane bagasse is an interesting source of cellulose and hemicellulose, polymers that can be used in the fermentative process for fuel alcohol production [1]. Although the industrial yeast *Saccharomyces cerevisiae *efficiently ferments hexoses, this yeast is unable to ferment pentoses such as xylose (present in hemicellulose hydrolysates) or the disaccharide cellobiose (present in cellulose hydrolysates). Thus, we have characterized the enzymes and transport systems involved in xylose and cellobiose fermentation by yeasts species isolated in rotten wood from several Brazilian ecosystems [2-4].

## Methods

The xylose fermenting yeasts *Spatahspora arborariae, S. passalidarum *and *Candida queiroziae *were grown on rich YP (2% peptone and 1% yeast extract) medium with 2% of glucose, xylose or cellobiose as carbon source. The xylose reductase activity was measured by monitoring the oxidation of NADPH or NADH, while the xylitol dehydrogenase activity was measured by monitoring the reduction NAD^+ ^or NADP^+ ^at 340 nm as described [5]. The intracellular β-glucosidase was assayed using permeabilized yeast cells using cellobiose or p-nitrophenyl- β -glucopiranosíde (pNPβG) as substrates. The active proton co-transport with xylose or cellobiose was determined using a pH-meter as previously described for other yeast sugar-H^+ ^symporters.

## Results and conclusion

Our results showed that the fermentation of xylose, cellobiose and glucose is a variable trait in the yeasts isolated from rotten wood. *S. arborariae *and *S. passalidarum *fermented xylose better than glucose probably due to a xylose reductase with significant activity (*K*_m _of 10-18 μM and *V*_max _of 0.38-0.50 U mg^-1^) not only with NADPH, but also with NADH as cofactor, while the xylitol dehidrogenase was totally dependent on NAD^+ ^(*K*_m _of 100 μM and *V*_max _of 0.25 U mg^-1^). Our results also show that *S.arborariae *has a H^+^-xylose cotransport system with low affinity and high capacity (*K*_m _of 25 mM and *V*_max _of 35 nmol mg^-1 ^min^-1^) for the sugar. While this last yeast could not ferment cellobiose, only half of the *S. passalidarum *strains could ferment this sugar due to the presence of an intracellular β -glucosidase as already described for *C.queiroziae *[3], a yeast specie which has an high affinity H^+^-cellobiose cotransport system (*K*_m _of 1,5 mM and *V*_max _of 19 nmol mg^-1 ^min^-1^). Thus, the xylose and cellobiose fermenting yeasts characterized in this work may constitute an interesting source of enzymes and/or transporters (and their corresponding genes) with more appropriate characteristics for the fermentation of these sugars, that may the expressed in industrial yeasts aimed at optimizing bioethanol production in Brazil.
